# Conserved expression of transposon-derived non-coding transcripts in primate stem cells

**DOI:** 10.1186/s12864-017-3568-y

**Published:** 2017-02-28

**Authors:** LeeAnn Ramsay, Maria C. Marchetto, Maxime Caron, Shu-Huang Chen, Stephan Busche, Tony Kwan, Tomi Pastinen, Fred H. Gage, Guillaume Bourque

**Affiliations:** 1 Department of Human Genetics, McGill University, Dr Penfield Avenue, Montreal, H3A 1B1 Canada; 2 Lab of Genetics, Salk Institute for Biological Studies, 10010 N Torrey Pines Rd, La Jolla, CA 92037 USA; 3 McGill University and Genome Quebec Innovation Centre, 740 Dr Penfield Avenue, Montreal, H3A 1A4 Canada

**Keywords:** Transposable elements, Long non-coding RNAs, Induced pluripotent stem cells

## Abstract

**Background:**

A significant portion of expressed non-coding RNAs in human cells is derived from transposable elements (TEs). Moreover, it has been shown that various long non-coding RNAs (lncRNAs), which come from the human endogenous retrovirus subfamily H (HERVH), are not only expressed but required for pluripotency in human embryonic stem cells (hESCs).

**Results:**

To identify additional TE-derived functional non-coding transcripts, we generated RNA-seq data from induced pluripotent stem cells (iPSCs) of four primate species (human, chimpanzee, gorilla, and rhesus) and searched for transcripts whose expression was conserved. We observed that about 30% of TE instances expressed in human iPSCs had orthologous TE instances that were also expressed in chimpanzee and gorilla. Notably, our analysis revealed a number of repeat families with highly conserved expression profiles including HERVH but also MER53, which is known to be the source of a placental-specific family of microRNAs (miRNAs). We also identified a number of repeat families from all classes of TEs, including MLT1-type and Tigger families, that contributed a significant amount of sequence to primate lncRNAs whose expression was conserved.

**Conclusions:**

Together, these results describe TE families and TE-derived lncRNAs whose conserved expression patterns can be used to identify what are likely functional TE-derived non-coding transcripts in primate iPSCs.

**Electronic supplementary material:**

The online version of this article (doi:10.1186/s12864-017-3568-y) contains supplementary material, which is available to authorized users.

## Background

It has been shown that the majority of the human genome is transcribed but that most of the resulting RNA products do not encode for proteins [1, 2]. Notably, some of the long non-coding RNAs (lncRNAs), defined as non-coding transcripts longer than 200 base pairs, are known to play important biological roles [3–5]. Moreover, it has been shown that an important source of lncRNA sequences are transposable elements (TEs), which make up about 50% of the human genome [6]. Specifically, it was reported that many lncRNAs are initiated in TEs and that about 75% of them have at least one exon overlapping a TE [7]. Actually, it has also been proposed that TE-derived sequences in lncRNAs may provide pre-formed functions to these transcripts [7, 8].

One example of a TE-derived lncRNA is lnc-RoR, a transcript implicated in the modulation of reprogramming of human iPSCs [9], which initiates in the human endogenous retrovirus subtype H (HERVH) [7]. HERVH is one of the most abundant human endogenous retroviral families in the human genome with about 1000 copies [10] and recent studies have found that HERVH instances are highly and specifically expressed in human embryonic stem cells (hESCs) [11, 12]. Moreover, it was shown that the expression of these TE-derived lncRNA transcripts helps define the naive stem-cell state [13] and knockdown experiments confirmed that this expression is essential for the maintenance of pluripotency in human stem cells [14]. HERVH-derived lncRNAs are probably not the only TE-derived transcripts involved in stem cell pluripotency, as knockdowns of several lncRNAs result in exit from the pluripotent state [15].

TE sequences are repeated throughout the genome because of their ability to replicate and insert into genomic DNA. There are several mechanisms through which this replication can occur, which defines the broadest classification of TEs: DNA, ERV/LTR, LINE and SINE [16]. TEs have freqently been ignored in genomic studies because of their repetitive nature, which makes them more difficult to deal with computationally, but their impact has gained recognition as many of them have been shown to be involved in the formation of new transcripts [13, 14] and regulatory innovations [17–19]. Although some TEs have a well characterized function in their host, such as in pluripotency [14] and X-chromosome dosage compensation [20], the majority of them have no known function. Comparative genomic studies have been shown to be a powerful way to identify functional elements in the genome [21]. An early study of this type looking at TEs noted that conserved repeats were preferentially located near genes that were associated with development and transcription regulation [22]. More recent studies focusing on lncRNAs found that these transcripts were expressed in a highly tissue specific manner [23] and even more so than protein coding genes [24]. Research examining cross-species lncRNA expression found that this high level of tissue specificity was well conserved in primates, but not in more distant species [23]; and that about 30% of lncRNAs were primate specific [25].

While a number of studies have examined the expression and evolution of non-coding RNAs in mammals [26, 27], none have focused on primate non-coding RNAs and on the link between TEs and lncRNAs. To identify TE-derived non-coding RNAs with important genomic functions, such as HERVH, we posited that cross-species expression data would be informative. In this context, and because of the rapid evolution of the lncRNA repertoire, we generated RNA-seq data from induced pluripotent stem cells (iPSCs) of several primate species: human (Homo sapiens), chimpanzee (Pan troglodytes), gorilla (Gorilla gorilla gorilla), and rhesus (Macaca mulatta). Using this resource, we looked for TE-derived non-coding transcripts with conserved expression profiles.

Using the same RNA-seq data, we also developed an iPSC-specific lncRNA catalogue for human, chimpanzee, gorilla, and rhesus. With this catalogue, we were able to identify repeat families that have contributed the most DNA to primate iPSCs lncRNAs. We were also able to identify several TE-derived lncRNAs, such as HERVH, that are well conserved in terms of having a large number of orthologous instances that are expressed in human and in some of the non-human primate (NHP) species. Several of these well conserved TE-derived lncRNAs have not been characterized before and could be novel functional transcripts.

## Results

### Conservation of TE instances in primate genomes

We first wanted to examine the conservation of TE instances between human and NHP species before looking at any expression data. By conservation here we mean TE instances occurring at orthologous locations in the different primate species and not having been lost in one of the genomes. To do so, we performed various pairwise comparisons to determine how many TEs occur in corresponding genomic locations. First, TE annotations were generated for human, chimpanzee, gorilla, and rhesus using the RepeatMasker software [28] (Methods). As expected, the repeat catalogues were found to be comparable in size, with each species having between 4.2-4.5 million TE instances (Additional file 1: Table S1). Next, conserved TEs were identified by determining if they existed at orthologous genomic locations using the UCSC LiftOver tool [29]. TEs were labeled as putatively conserved if they could successfully LiftOver between species (Methods). We restricted the analysis to TEs which did not overlap coding regions, as we are interested in the contribution of TEs to non-coding transcripts.

**Fig. 1:**
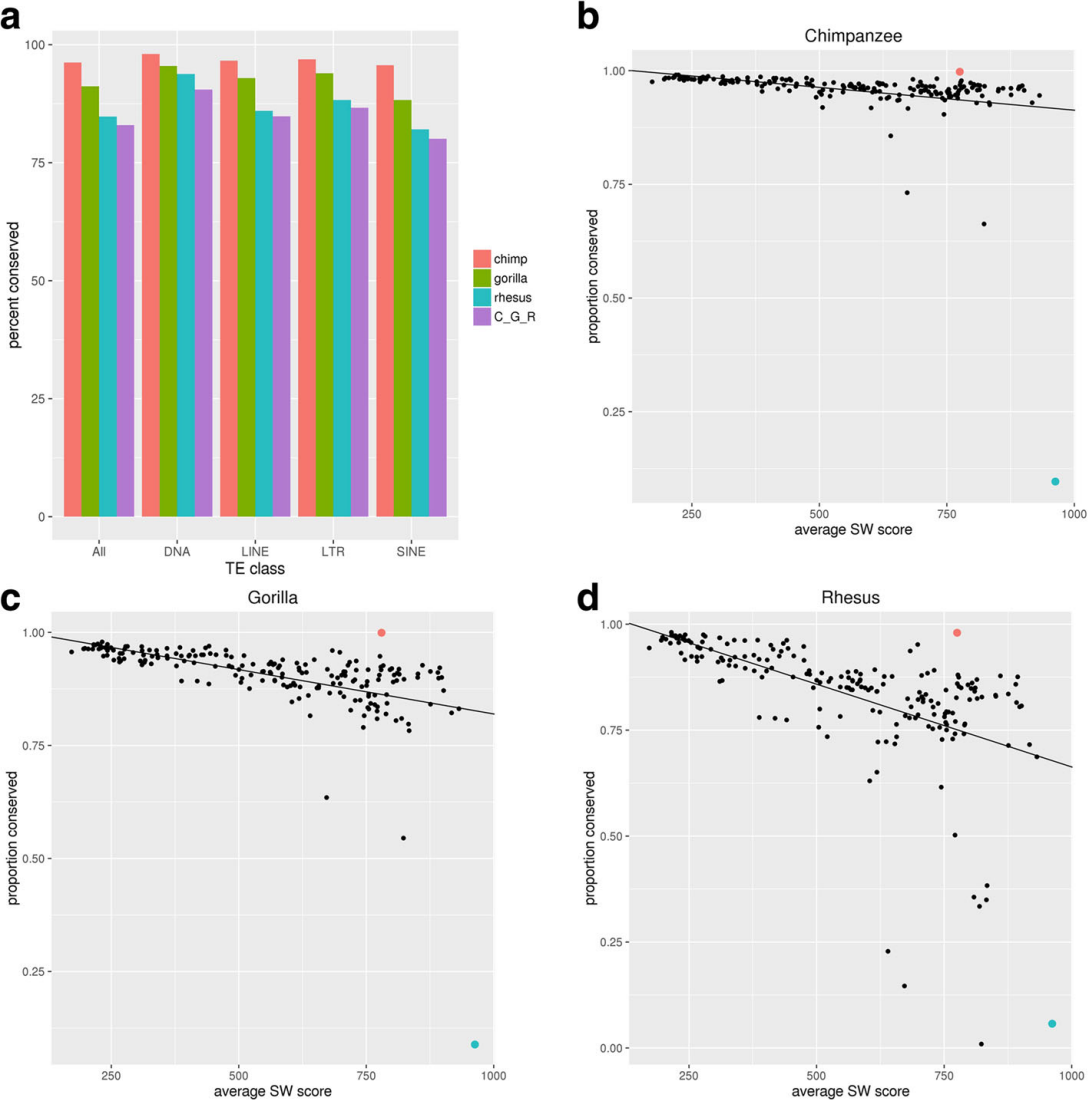
**a** Percent of all human TEs which are conserved in NHP grouped by TE class. C_G_R indicates TEs conserved in all 3 NHP species. **b** The proportion of instances in each TE family that are conserved in Chimpanzee (y-axis) relative to the average sequence identity score of the family (x-axis). Sequence similarity to the family’s consensus sequence is shown as a scaled Smith-Waterman (SW) score. This is used as a surrogate for the age of the TE family. Older TEs are on the left, and newer insertions are to the right. TE families which inserted more recently are less conserved than older TEs. TE families were filtered for those with more than 30 expressed instances in human. tRNA-Asn-ACC in *red*, AluYa5 in *blue*. **c** The same in Gorilla (**d**) The same in Rhesus

Overall we found that more than 90% of repeat instances across all major repeat families were conserved between human and chimpanzee or gorilla, about 85% were conserved in rhesus and over 80% were conserved across all analyzed primate species (Fig. 1 a). This high conservation between human and NHPs can be explained by their relatively recent divergence. We note that repetitive sequences are sometimes missing from assemblies of lower quality, such as the ones we are using here for the NHP, and so these estimates should be taken as lower bounds.

We can verify our approach to identify conserved TE instances by using the well-characterized HERVH repeat family. Using the above annotation strategy, human, chimpanzee, and gorilla were found to have 1266, 1157, and 1276 HERVH instances, respectively. This is consistent with recent estimates that indicated that approximately 1000 insertions occurred in higher primates [30]. Rhesus has fewer HERVH instances (742), which can by explained by the fact that this family expanded after the divergence from rhesus. We can also examine the regions of the HERVH consensus sequence that are being attributed to these insertions (Additional file 1: Figure S1). The patterns observed are consistent with the known evolutionary history of HERVH, with a large expansion that took place after the split from rhesus and that contain a deletion in what used to correspond to the ENV gene [31]. To verify that LiftOver is identifying orthologous sequences accurately, we examined the TE annotation of conserved human HERVH instances in NHPs. We found that of the human HERVH instances that successfully lift to chimpanzee and gorilla over 90% were independently annotated as HERVH in that species (Additional file 1: Table S2), which further supports the accuracy of the methodology.

Next, we were interested in TE conservation at the level of families. Using a method previously described to estimate the age of each family [32] (Methods), we observed that the older TE families were more conserved than recent ones (Fig. 1 b-d and Additional file 2: Table S3). This is to be expected given that the younger families tend to be associated with more recent expansions whose instances can be absent from the other genomes. This downward trend was most prominent in rhesus, the most distant analyzed species. In this analysis, one outlier TE family appears to be conserved more than expected based on its family age (shown in red in Fig. 1 b-d). This TE family is tRNA-Asn-AAC, a repetitive non-coding gene which produces a tRNA. It is classified as a TE due to its repetitive nature, but because it is a functional tRNA conserved across all mammalian cells, its high level of conservation can be explained. This TE family has 46 instances in the human genome, not overlapping annotated coding regions, 45 of which are conserved in the NHPs we studied. In contrast, AluYa5 (shown in blue in Fig. 1 b-d) is a very recent family which is poorly conserved between human and rhesus. The AluY family is still active in the human genome [33]. AluYa5 has 2290 instances not overlapping coding regions, only 131 (5.7%) of which are annotated as putatively conserved in rhesus.

### TE expression is conserved between human and non-human primates

**Fig. 2:**
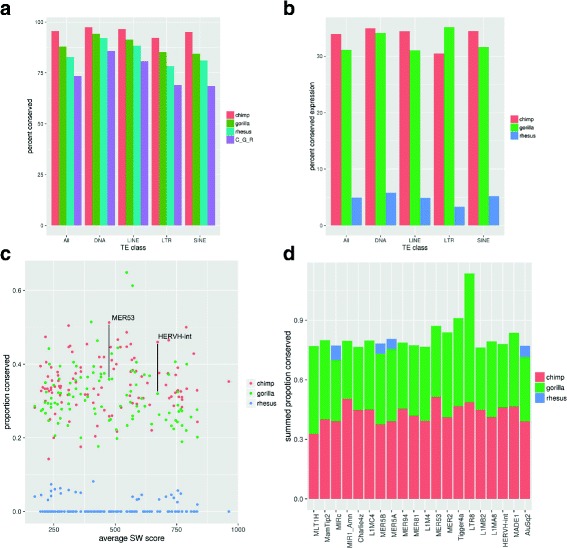
**a** Percent of all TEs expressed in human which are conserved in NHPs, grouped by class. C_G_R indicates TEs conserved in all 3 NHP species. **b** Percent of human expressed TEs which are conserved in terms of sequence and expressed in NHPs. **c** TE families plotted by average sequence identity and proportion conserved in NHPs. Y axis specifies the proportion of human expressed TEs which are also expressed in NHPs. Average sequence divergence is used as a surrogate for age, with older TEs on the left. This plot shows only families with 30 or more expressed TEs and 10 or more conserved in the target species. **d** Top 20 most conserved families (sequence and expression) when their conservation is summed across all 3 NHP species

Next, we wanted to examine TE instances with conserved expression profiles in primate iPSCs. We generated RNA-seq data from four primate iPSC cell lines: 3 human, 1 chimpanzee, 2 gorilla, and 1 rhesus (Methods and Additional file 1: Table S4). We found that TE instances that had an expression level of at least 1 RPKM in human and did not overlap protein coding genes were conserved, in terms of having an orthologous locus in NHP, at slightly lower levels than TEs in general (Fig. 2 a). This is probably because expressed TEs tend to be younger than non-expressed TEs (Additional file 1: Figure S2). Still, over 70% of expressed human TEs were found to have an orthologous sequence in all NHP species studied. Next, when we looked to see which of these conserved TE instances were also expressed in the other species, we observed that about 33% of them had orthologous expression in chimpanzee and slightly less in gorilla (Fig. 2 b). This type of expression conservation dropped dramatically when rhesus was taken into account. Clearly although many TE instances have been retained in both human and rhesus, they are also under different expression controls in the two species. In this context, TEs that do exhibit expression conservation between human and rhesus are especially interesting since expression has been conserved over a long evolutionary time period.

To further examine the statistical significance of the conservation of expression at the level of individual repeat families, we performed a hypergeometric test (Methods). We removed small repeat families, Simple Repeats, and low complexity repeats from this analysis since they are more prone to biases [32]. Looking at expression conservation based on the level of sequence conservation of the family did not reveal any unusual patterns (Additional file 1: Figure S3 and Additional file 2: Table S3). The TE families that had the highest proportion of instances with conserved expression between human and chimpanzee are shown in Table 1 (see also Additional file 3: Table S5). In particular, the MER53 repeat family had the largest percentage of TEs with such conserved expression (51%, *p*-value = 9.61∗10^−27^, Fig. 2 c). Notably, this TE family has previously been shown to be the source of a placental-specific family of miRNAs [34], the miR-1302 family, and to be expressed in human embryonic stem cells [35]. The instances of MER53 that are highly expressed and conserved among primates do not overlap with annotated miR-1302 transcripts, however the annotated transcripts are only ones that have been experimentally validated. The fact that the expression of the MER53 instances is highly conserved suggest a potential role for this family in both human and chimpanzee iPSCs.

**Table 1:** TE expression conservation between human and chimpanzee iPSCs

Family	Class	Total	Human	Chimpanzee	Proportion	*p*-value
			expressed	expressed		
MER53	DNA	5308	39	20	0.51	9.61∗10^−27^
MIR1_Amn	SINE	9495	113	57	0.50	1.71∗10^−71^
AluSg7	SINE	5780	50	25	0.50	6.81∗10^−35^
LTR8	LTR	2516	37	18	0.49	1.81∗10^−25^
L2d	LINE	19063	135	64	0.47	2.32∗10^−84^
Tigger4a	DNA	3242	45	21	0.47	2.41∗10^−20^
MADE1	DNA	7634	86	40	0.47	7.59∗10^−51^
HERVH-int	LTR	1266	50	23	0.46	9.26∗10^−15^
MER94	DNA	4884	33	15	0.45	2.17∗10^−21^
L1MC1	LINE	7375	33	15	0.45	8.19∗10^−22^
L1MC4	LINE	12920	109	49	0.45	4.17∗10^−64^
L1MB2	LINE	4967	38	17	0.45	1.49∗10^−20^
Charlie4z	DNA	5255	47	21	0.45	1.38∗10^−27^
L1MB8	LINE	9006	56	25	0.45	7.32∗10^−34^
L1M2	LINE	6281	56	25	0.45	4.05∗10^−32^
OldhAT1	DNA	1897	30	13	0.43	5.22∗10^−16^
AluYk3	SINE	5421	42	18	0.43	5.12∗10^−24^
MER81	DNA	3551	31	13	0.42	4.35∗10^−18^
L1MC3	LINE	6596	31	13	0.42	1.36∗10^−17^
MER1B	DNA	5060	36	15	0.42	2.05∗10^−20^

Here we examine only large families (≥100) from the main repeat classes (DNA, SINE, LINE, LTR). The table is sorted by the proportion of human expressed TEs which are conserved in chimpanzee. Only repeat families with at least 30 expressed instances in human are shown

Another well characterized example that appears in Table 1 is HERVH with 50 instances expressed in human, which is comparable to what was found in previous studies [11, 12, 14]. Notably, we found 23 orthologous instances also expressed in chimpanzee, which makes it one of the families with the highest expression conservation(46%, *p*-value = 9.26∗10^−15^). The combined expression conservation of human expressed TEs in NHP also revealed HERVH as one of the top conserved TE families (Fig. 2 c-d). We observed similar numbers of HERVH instances expressed in human, chimpanzee, and gorilla (around 5%) and very few instance expressed in rhesus (Additional file 1: Figure S4). The differences we observe between human, chimpanzee, and gorilla HERVH expression are fairly small considering the size of the family and the number of instances expressed, which are both fairly high compared to most TE families. The low number of expressed HERVH in rhesus is expected since rhesus has fewer copies of this repeat family. The high conservation of expression of HERVH suggest that the HERVH-lncRNA function revealed in human [13, 14] arose before the divergence of gorilla.

Finally, it has been noted that some Alu repeats exhibits mobilization in iPSCs [36] and a few Alu families are found to have high expression conservation (Table 1). However, most of the other TEs on this list have limited literature describing them, so further validation would be needed to determine if they play a role in primate iPSCs.

### iPSC-specific lncRNAs in primates frequently overlap transposable elements

Several studies have shown that a large portion of lncRNA sequence is made up of TEs [7, 24]. Since we were able to identify a number of TE families with significant conservation of expression between primate species we were interested to see if some of these TEs also contributed to lncRNA transcripts with conserved expression. Publicly available lncRNA catalogues for non-human primates are not nearly as complete as the human lncRNA catalogues. For this reason we created iPSC-specific lncRNA annotations for each species based on our iPSC RNA-seq data using the FEELnc pipeline [37] (Additional file 1: Figure S5, Methods and Additional file 1: Table S6). Briefly, using the transcripts assembled from the RNA-seq data, we filtered the transcriptome for protein coding genes, mono-exonic transcripts, and any transcripts with protein coding potential. We chose to remove mono-exonic transcripts from this analysis because it was not possible to avoid systematic false positives in that category without extensive manual curation.

**Fig. 3:**
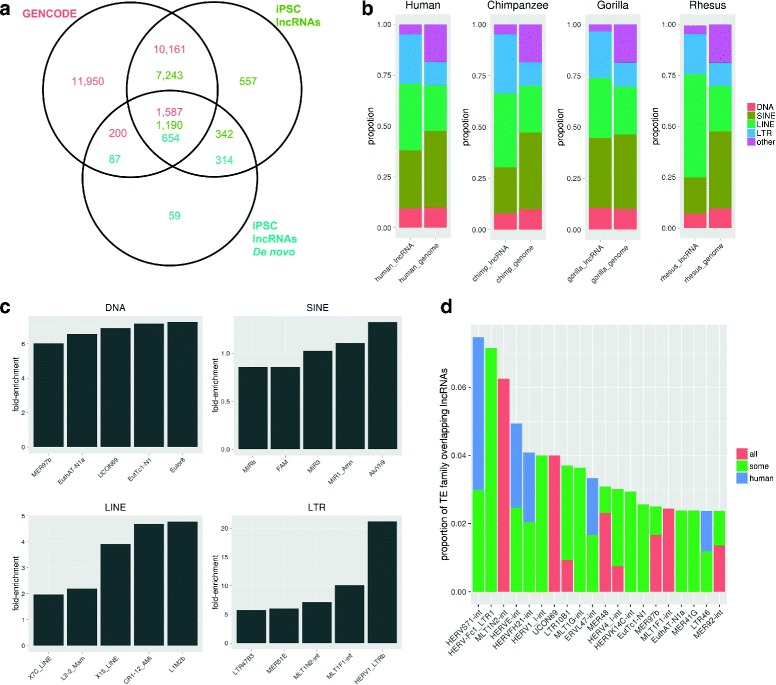
**a** The intersection of 3 lncRNA annotations: iPSC with guide annotations, iPSC without guide, and GENCODE lncRNAs expressed in iPSCs. Over 90% of FEELnc human lncRNAs overlap with GENCODE lncRNAs. **b** The proportions in each primate lncRNA annotation compared to the genomic TE proportion. Only lncRNAs that overlap TEs are included in these proportions. **c** The proportion of human lncRNA sequence made up by TE families normalized by proportion of the genomic sequence made up by each family. The top 5 families from each of the 4 main classes are shown. **d** TEs that occur most frequently in human iPSC lncRNAs normalized by the size of each TE family. Only families with more than 10 members are shown. *Red* represents lncRNAs which are conserved in all 4 primate species. *Green* are those conserved in 1 or 2 other NHPs. *Blue* are human specific lncRNAs

We validated this automated method for annotating lncRNAs using RNA-seq data by comparing it against GENCODE. Our human annotation contains 9,332 lncRNAs, 90.8% of which overlapped the GENCODE catalog (green values in Fig. 3 a). After performing expression analysis on GENCODE lncRNAs with our iPSC data we noted that about half of the iPSC expressed GENCODE lncRNAs did not appear in our annotation (data not shown). However, the majority of these missing transcripts are either overlapping protein coding genes or are mono-exonic transcripts, which is consistent with our selection criteria.

The FEELnc method for identifying lncRNAs that uses gene annotation as a guide for transcriptome assembly with Cufflinks will perform differently depending on the quality of that annotation. The smaller size of our lncRNAs catalogs in NHP (Additional file 1: Table S6) is likely due to having fewer replicates but also a reflection of the more limited annotation in those species. When we applied the FEELnc method without such guide annotation we obtained approximately the same number of lncRNAs in human, chimpanzee, and gorilla (Additional file 1: Table S6 and blue values in Fig. 3 a). Such de novo lncRNA annotation for rhesus resulted in very few lncRNAs detected. This seems to be due to a number of factors including: poor genome assembly and fewer aligned reads for rhesus. Since removing the guide annotation during the transcriptome assembly reduces the number of human lncRNA transcripts to a number comparable to chimpanzee and gorilla, we suspect the quality of the annotation to be the main cause of discrepancy in the lncRNA catalogue size for the different primate species.

Finally, for each lncRNA catalogue, we examined the proportion of the lncRNAs which had at least 10% of one exon overlapping a TE (Methods). In human we found that about 73% of the lncRNAs overlapped a TE (Additional file 1: Table S6), which is consistent with previous studies which examined human lncRNA catalogs [7, 24]. We found this proportion to be fairly consistent across all primate species, and also in the de novo lncRNAs cataglogues.

### Some TE-derived lncRNAs have conserved expression

Finally, we were interested in the expression conservation of the primate iPSC lncRNAs. This analysis was done in conjunction with the TE annotations to identify TE-derived lncRNAs whose expression is conserved. Using LiftOver to convert human lncRNA coordinates to NHP, we found that the majority of lncRNAs were conserved between human and NHPs (Table 2). For instance, of the 9,332 iPSC-specific lncRNAs in human, 7,226 have orthologous positions in chimpanzee. Next, we looked at which of these conserved lncRNAs were also expressed in the other species (Methods and Table 2). We found, in chimpanzee, that of the human lncRNAs that LiftOver, 40% were also expressed in chimpanzee.

**Table 2:** Conservation and TE contribution to human iPSC lncRNAs

Lift human to _	LiftOver	LiftOver, TEs	Expressed in target species	LiftOver, Expressed, TEs
Chimpanzee	7479	5175 (69.19%)	2981 (39.86%)	2103 (28.12%)
Gorilla	6709	4707 (70.16%)	2086 (31.09%)	1465 (21.84%)
Rhesus	6208	4550 (73.29%)	1527 (24.60%)	1351 (21.77%)

Column 2: The number of human lncRNAs which lift to each NHP (out of 9332 human lncRNAs). Column 3: The number of lifted lncRNAs which overlap TEs in the target species. Column 4: Lifted human lncRNAs that are expressed in the target species. Column 5: Lifted and expressed lncRNAs that overlap TEs

Next, we labeled each lncRNA transcript with the TE it overlapped the most, whenever such an overlap corresponded to at least 10% of one of its exons (Methods). With such an annotation, we found no difference in the proportion of TEs contributing to all human lncRNAs and human lncRNAs with an orthologous NHP lncRNAs (Additional file 1: Figure S6). In the human lncRNAs, we observe a clear enrichment of LTRs and LINEs and a depletion of SINE elements as compared to the genomic proportion of these TEs (Fig. 3 b). These patterns of enrichment and depletion of TE classes in lncRNAs are consistent with what has been observed in other studies [7, 24]. Notably, we are now also able to observe the same patterns in NHP lncRNA catalogues (Fig. 3 b).

When we investigated which TE families contributed the most sequence to lncRNA transcripts in human we noted that HERVH was one of the top contributors from the LTR class (Additional file 1: Figure S7). We also found that MLT1J was the member of the LTR family which contributed the most absolute sequence to human lncRNAs. Previous studies have shown that MLT1J harbors transcription factor binding sites for YY1 [38] and YY1 is known for repressing and activating a number of different promoters. In this way, the transposon MLT1J may have contributed YY1 binding sites to the genome, like the OCT4 binding sites in the promoter sequences of HERVH [18], thus allowing the expression of its internal sequence to be maintained. When the amount of lncRNA sequence contributed by TEs is normalized by the genomic size of each family, it becomes clear that some DNA and LTR families contributed more than expected by chance (Fig. 3 c).

In the final part of this analysis we determined human lncRNAs that are conserved in NHPs, and used this information to determine which TE families contribute the most sequence to these conserved transcripts. This analysis revealed TE families that overlap most frequently with human lncRNAs and how many of these transcripts are conserved in all, some, or none of the NHPs (Fig. 3 d, Additional file 1: Figure S8). We identified several subfamilies of MLT1 that contributed large amounts of sequence to these conserved transcripts (Additional file 1: Figure S8), even when we took into account the genomic size of these subfamilies (Fig. 3d). For instance, two MLT1 subfamilies contribute the most to conserved lncRNA: MLT1N2 and MLT1F1. The MIRb family overlapped most frequently with human lncRNAs and is very well conserved in NHPs. While this TE family has been associated with non-coding variants of the CHRM2 gene [39], which is involved in neuron function, it is not widely recognized as contributing to lncRNAs.

## Discussion

In this work, we utilized RNA-seq data from primate iPSCs to identify TEs producing potentially functional non-coding transcripts. We were able to characterize a number of TE families that are well conserved among human, chimpanzee, gorilla, and rhesus. In particular, we were able to identify a number of TE families with conserved expression (Fig. 2, Table 1). The conservation of TE expression across several primate species is an indicator of potential function and some of the TE families that we identified in this way, most notably HERVH [13, 14], have an already well-characterized biological function. Others, like MER53, are potentially interesting due to their conserved expression profile (Fig. 2 c-d) and the fact that they are the source of a placental-specific family of miRNAs [34]. The family Tigger4a, from the DNA repeat class, also had high expression conservation when summed across all primate species (Fig. 2 d) and highly significant *p*-value when tested for enrichment of expressed instances in conserved TEs between human and chimpanzee (Table 1). Tiggers are part of the mariner/Tc1 family originally discovered in drosophila [40], but not much is known about the potential function of Tiggers in human.

In this study, we also utilized the RNA-seq data to create iPSC-specific lncRNA annotations for these four primate species. Using these catalogues we identified TE families which contributed the most lncRNA sequence in human and non-human primates. We also noted TE families that most frequently occur in conserved lncRNA transcripts. Some of the TE families that appeared several times in our analysis are MLT1-type families (Figs. 2 d, 3 c-d, Additional file 1: Figure S7). Previous studies have shown that at least one of these families, MLT1J, harbors transcription factor binding sites for YY1 [38], which could explain why its expression has been maintained. We also noted that several Tigger families appear to have also contributed a significant amount of sequence to human lncRNAs (Additional file 1: Figure S7). Large-scale cDNA studies [41, 42] have identified several putative, Tigger-derived proteins, but based on these results we believe some members of the Tigger family may have also contributed important sequences to the primate lncRNA repertoire. These examples are just a few of the TE families that were highlighted in this study and potentially play important roles as non-coding transcripts in primate stem cells.

## Conclusions

Our study focused on conserved expression of TE-derived lncRNAs, but many of the conserved TEs that were identified do not overlap lncRNAs. It is possible that TEs with conserved primate expression are being transcribed as by-products of an exapted enhancer or promoter, or as other transcripts like miRNAs. In a way, our list of potentially interesting TEs could be useful to explore the impact of TEs on other genomic functions. Several of the conserved TE families that were discussed here are not well characterized and would be good candidates for further experimental validation. In particular, experiments such as knockdowns and subcellular localization could be used to better determine their biological function.

## Methods

### Generating transposable element annotations

Using the genome builds hg19, panTro4, gorGor3, and rheMac3 for human, chimpanzee, gorilla, and rhesus, respectively, we generated TE annotations. TEs were annotated for each genome using RepeatMasker version 4.0.510 [28], RepBase Library version 20140131 [43], and the UCSC script extractNestedRepeats.pl [29]. We created our own TE annotation for this study to ensure consistency between assemblies. RepeatMasker was run using the following options: species - to identify the appropriate species, slow - to increase accuracy, and parallelize - to decrease runtime. After joining all the chromosome output files the results of RepeatMasker were passed to extractNestedRepeats.pl [29], which is a script available on the UCSC genome wiki page about the generation of UCSC repeat tracks. This is the method used to create the NestedRepeats track in the UCSC genome browser. extractNestedRepeats.pl uses the ID values given by RepeatMasker to join repeat instances that likely belong to the same insertion. In its original format the script only outputs repeats which can be merged, so we modified it to also output any repeats that did not require joining. This creates a complete track of repeat instances. The RepeatMasker output also includes information about the divergence of each TE from its family’s consensus sequence. This is determined using a Smith-Waterman alignment of each TE against its consensus, this score is averaged and transformed to a 0-1000 scale by extractNestedRepeats.pl for shading in the UCSC genome browser. In this study the consensus sequence is taken as an approximation of the ancestral sequence. Since TE sequence typically degrades over time we can estimate age from sequence divergence [32]. The age of whole TE families was determined by averaging the Smith-Waterman score of all family members.

### Identifying orthologous transposable elements

We identified orthologous regions for human TEs in non-human primates (NHPs) using the UCSC LiftOver utility [44]. This program converts genome coordinates between genome assemblies. It uses a pairwise alignment of the genomes (chain files) generated by UCSC to convert given intervals between species. An important option in LiftOver is the -minMatch, or the minimum ratio of bases that must remap. 10% is the default minimum value on the UCSC genome browser when lifting between different species’ genome builds. In order to confirm the option -minMatch 0.1 is appropriate we compared the lifted annotation with the RepeatMasker annotation in the target species at several cut off values. In particular, we examined HERVH annotations since we can infer the expected conservation based on the evolutionary history of its insertions. HERVH was lifted to each genome, and then verified to be annotated as HERVH by RepeatMasker in the target genome build. In all 3 primate species we found that at least 85% of the lifted HERVH are correctly, independently annotated as HERVH (Additional file 1: Table S2). This provided some evidence that a rate of 10% remapping is an appropriate value to accurately lift repeat intervals.

### Primate iPSC cell culture

The Human iPS cell lines WT-33, ADRC-40 and WT-126 were previously described [45], as was the method for generating iPSC cell lines for non-human primates [46]. Briefly, Fibroblasts from P. troglodytes (chimpanzees: PR00818), G. gorilla (gorilla: PR00053 and PR00075), and M. mulatta (rhesus) were from Coriell Cell Repositories (NJ). All fibroblasts were cultured in MEM (Invitrogen) supplemented with 10% FBS (HyClone Laboratories). Retroviral vectors expressing OCT4 (also known as POU5F1), MYC, KLF4 and SOX2 human cDNAs from Yamanaka’s group [47] were obtained from Addgene. Recombinant viruses were produced by transient transfection in 293T cells (ATCC - CRL-3216), as previously described [48]. Two days after infection, cells were plated on mitotically inactivated mouse embryonic fribroblasts (Chemicon) with human ES cell medium. after 2-4 weeks, iPSC cell colonies were picked manually and directly transferred to feeder-free conditions on matrigel-coated dishes (BD) using mTeSR1 (StemCell Technologies). Established iPS cell colonies were kept in feeder-free conditions indefinitely, and passed using mechanical dissociation. Embryoid-body-mediated differentiation in suspension was carried out for 10 days in the absence of growth factors.

iPSC clones continuously expressed pluripotency markers, retained undifferentiated morphology in culture, and maintained a normal karyotype. After embryoid body (EB)-mediated differentiation in vitro, clones contained tissue derivatives from the three embryonic germ layers and down-regulated expression of pluripotency markers.

### RNA-seq data generation

Three human, 1 chimpanzee, 2 gorilla, and 1 rhesus cell lines were generated and sequenced. RNA was isolated with miRNeasy Mini Kit (Qiagen) and 500ng of RNA were used to prepare libraries using Illumina TruSeq Stranded Total RNA Sample preparation kit following manufacturers directions. Quality control was performed using Bioanalyzer and samples were sequenced on Illumina HiSeq2000, 100bp paired-end reads. Raw reads were trimmed (quality: phred33 ≥30 and length n ≥32), adapters were removed (using Trimmomatic V.0.32 [49]) and reads were aligned to the hg19 human reference (Tophat v.2.0.10 [50] and bowtie v.2.1.0 [51]). We received samples in Trizol and then cleaned up the RNA. All the sequencing was done on Illumina HiSeq2000 100bp paired-end and indexed at 4-fold.

Gene expression clustering was done using protein coding genes that are orthologous between human and all 3 non-human primates. Human and non-human primate reads were mapped to hg19 and expression of protein coding genes was determined using HTSeq-count [52] and DESeq2 [53]. Clustering of top 1000 genes is shown in Additional file 1: Figure S9. PCA analysis of all orthologous protein coding genes was also performed (Additional file 1: Figure S10).

### Determining transposable element expression

Expression analysis was carried out on each species by first counting the coverage of RNA-seq reads over each TE. This step was performed using coverageBed from the BEDTools suite [54]. The counts were then normalized by library size and TE length using the method reads per kilobase of transcripts per million mapped reads (RPKM) [55]. Next, in the case of human and gorilla where we have biological replicates, we average the RPKM values over the replicates. Expressed TEs are defined as those with an RPKM of 1 or greater. The expression analysis was only conducted on TEs which do not directly overlap coding regions. In identifying TEs with conserved expression between species we only counted TEs which LiftOver and are annotated as a TE in the target species.

### Statistical analysis

We performed statistical analysis on the conserved sets of TE families. We utilized a hypergeometric test to determine whether the set of conserved TE sequences is enriched for being expressed in primate iPSCs. We wanted to know if there is an association between sequence conservation and expression. In a pairwise comparison of primate species this test gives us the probability of seeing *j* or more expression conserved TEs from a set of *i* TEs that are expressed in one of the species. Generally, the hypergeometric distribution is a discrete probability distribution describing the number of successful draws from a finite population without replacement.

We utilized a number of filtering techniques in order to ensure that our TE lists contain genuinely interesting families. To this end we removed very small TE families and simple repeats. Many of these very small families exhibited significant *p*-values when we examined for enrichment in conservation of expression between primates, but this is likely due to bias in the statistical test. Additionally these small families are much less likely to give rise to lncRNA transcripts, which is another reason why these particular types of TEs are not interesting in the context of this study. Moreover, simple repeats and low complexity regions frequently occur in high GC regions, which may affect their detection [19].

### Creating lncRNA catalogs

lncRNA annotations were generated for all four species using a combination of our own filtering techniques and a pipeline available online for annotating lncRNAs called FEELnc (Additional file 1: Figure S5) [37]. The first step of this analysis was to assemble the RNA-seq transcriptome for each species. This was done using Cufflinks [50] with default parameters and ensembl gene annotations as a guide. The guide annotations were obtained from UCSC for the genome builds hg19, panTro4, gorGor3, and rheMac3 for human, chimpanzee, gorilla, and rhesus, respectively. In the case of human and gorilla, for which we have biological replicate data, we also used Cuffmerge to merge the Cufflinks transcriptomes. Cuffmerge was also run with default parameters.

After we produced the iPSC transcriptome for each primate species we used FEELnc to filter out any transcripts that are not long non-coding. We generated our own filter file to remove any known transcripts other than lncRNAs. This includes protein coding genes, pseudogenes, and tRNAs among others(See Additional file 1: Table S7 for full biotypes list). This filtering step also removes mono-exonic transcripts. While there do exist some mono-exonic lncRNAs there are very few, and they are difficult to evaluate as true lncRNAs [24].

The next step of the pipeline removes transcripts with protein coding potential. To do this we used a version of the FEELnc pipeline which utilizes CPAT [13]. The optimal cutoff value for coding potential is calculated by CPAT using a training set of coding genes and intergenic regions. CPAT uses a 10 fold cross-validation on the training data to maximize sensitivity and specificity. Any transcripts with high protein coding potential are removed from our catalogues.

The method for annotating lncRNAs was evaluated by comparing our own human annotation against the GENCODE lncRNA annotation (version 19) [25]. After determining that the level of lncRNA detection was acceptable in human we used the same method to annotate lncRNAs in the non-human primates.

The lncRNA catalogues resulting from this pipeline had low numbers of transcripts annotated in NHP compared to human. We speculated that this was due to the fact that non-human primate genomes have poorer gene annotations compared to human. To test this we reran the pipeline without passing guide annotations to Cufflinks.

### Identifying conserved transcripts

After creating the lncRNA catalogues we used LiftOver to evaluate orthologous regions between the primate species. Based on the validation from TE LiftOver we again used 0.1 for the minimum ratio of bases that must remap. The conservation of lncRNAs was done based on our human annotation. We lifted lncRNAs from human to each of the 3 NHPs. We then performed expression analysis in non-human primates on the LiftOver lncRNAs to determine which are also expressed in NHP. RPKM values were calculated for each transcript using the Bioconductor package Rsubread. Reads were counted using featureCounts(), and normalized using rpkm() [56]. Expressed transcripts are defined as those with 1 RPKM or greater. For species with biological replicates this cutoff was required in all replicates to be deemed expressed.

### Annotating lncRNAs with TEs

After creating lncRNA catalogues for human, chimpanzee, gorilla, and rhesus we annotated the transcripts with TEs. This analysis uses two TE annotation methods. The first is a simple intersection between lncRNA exons and TEs. This was done using intersectBed from the BEDTools Suite [54]. We used the parameters -wao to output all intersection information (which features overlap and by how many base pairs), and -f 0.1 to require that at least 10% of an exon must overlap a TE for the intersection to be counted. An intersection of all TEs with our lncRNAs allowed us the look at which TEs contribute the most lncRNA sequence and examine the enrichment of specific families’ sequence contribution to lncRNAs. The second method of TE annotation is to label each lncRNA that overlaps TEs with a single TE (the sole element that overlaps, or the one that overlaps the most). This allows us to classify lncRNAs by TE family and evaluate which TE families occur most frequently.

## Additional files

Additional file 1 Supplementary Tables and Figures. (PDF 769 kb)

Additional file 2 Table of values for figures 1 and 2 and table 1. (TXT 1220 kb)

Additional file 3 Table of coordinates for table 1. (TXT 869 kb)

## Abbreviations

ERV: Endogenous retrovirus
HERVH: Human endogenous retrovirus subfamily h
hESC: Human embryonic stem cells
iPSCs: Induced pluripotent stem cells
lncRNA: Long non-coding RNA
NHP: Non-human primate
TE: Transposable element
