# Supplementary material for: Conserved expression of transposon-derived non-coding transcripts in primate stem cells
Source: BMC Genomics. 2017 Feb 28;18:214. doi: 10.1186/s12864-017-3568-y (PMC5331655; doi:10.1186/s12864-017-3568-y)
Supplement: Additional file 1 — Supplementary Tables and Figures. (PDF 769 kb) [file 12864_2017_3568_MOESM1_ESM.pdf]

Conserved expression of transposon-derived non-coding transcripts in primate stem cells

LeeAnn Ramsay, Maria C. Marchetto, Maxime Caron, Shu-Huang Chen, Stephan Busche, Tony Kwan, Tomi Pastinen, Fred H. Gage, Guillaume Bourque

1 Supplementary Figures

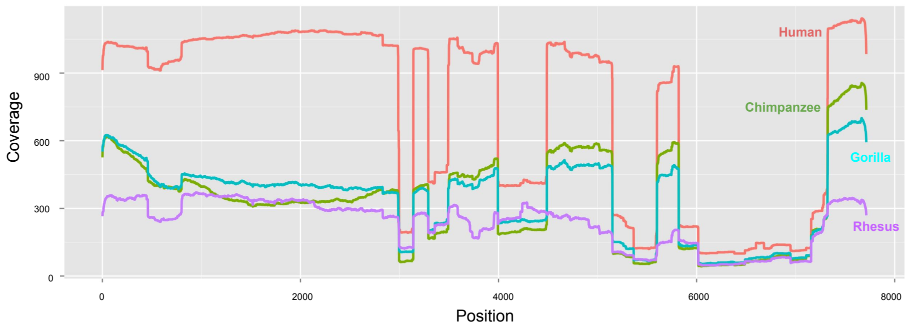

Figure S1: Observed HERVH sequence is consistent with its evolutionary history. X-axis is the consensus sequence for HERVH; integer values represent base pair locations. Y-axis is the number of HERVH instances which contain each base pair. At approximately base pair 6000 there is a large deleted segment. This is the ENV gene which was lost before major expansions of HERVH in the primate lineage.

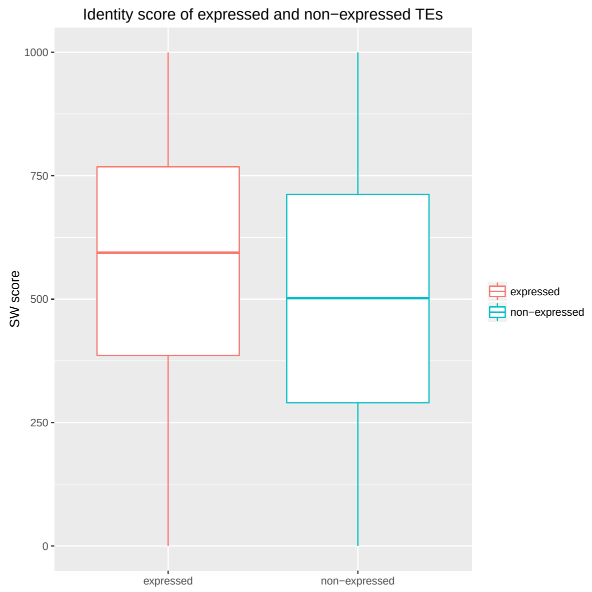

Figure S2: Distribution of identity scores for expressed and non-expressed TEs. We used scaled Smith-Waterman scores as a surrogate for age. Higher identity scores indicate younger TEs; more divergent sequences with lower scores are considered older. Expressed TEs are, on average, younger than non-expressed TEs. Expressed: RPKM ≥ 1, non-expressed: RPKM $= 0$.

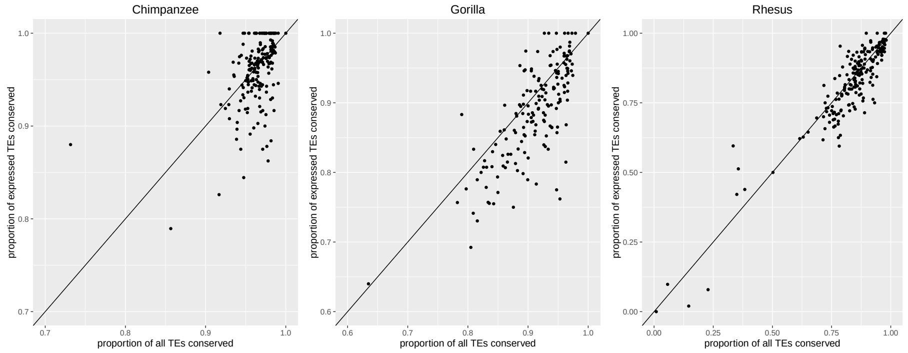

Figure S3: The proportion of entire TE families conserved between human and NHPs versus the proportion of human expressed TEs that are conserved. **(a)** Chimpanzee. **(b)** Gorilla. **(c)** Rhesus.

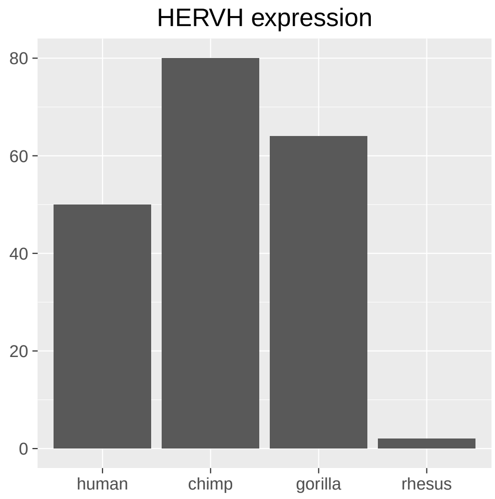

Figure S4: Number of HERVH expressed in each primates species.

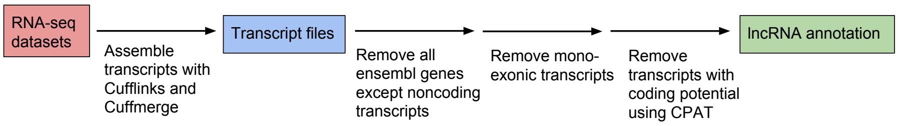

Figure S5: Ouline of the pipeline used to generate lncRNA catalogues.

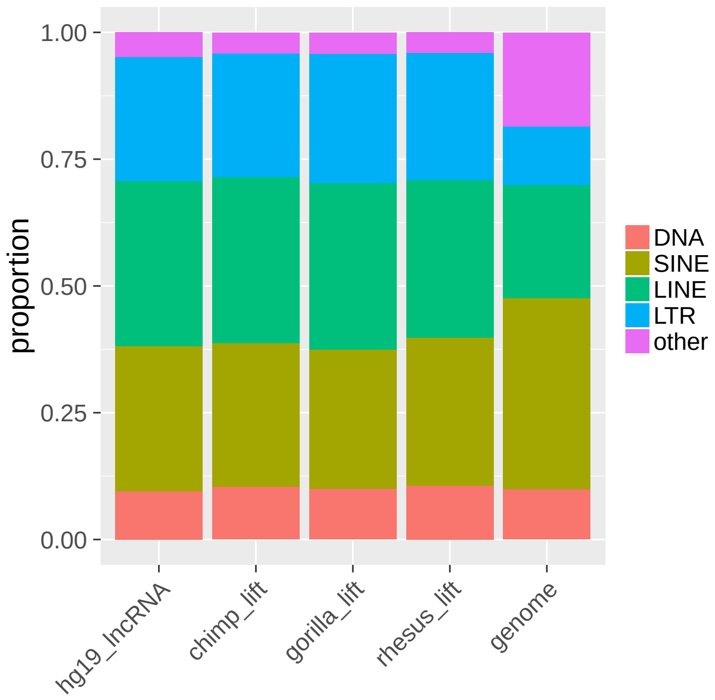

Figure S6: Proportion of lncRNAs labeled by TE class in human lncRNAs conserved in primates. Only lncRNAs that overlap TEs are included in these proportions. Rightmost bar is the genomic proportions of TEs in human.

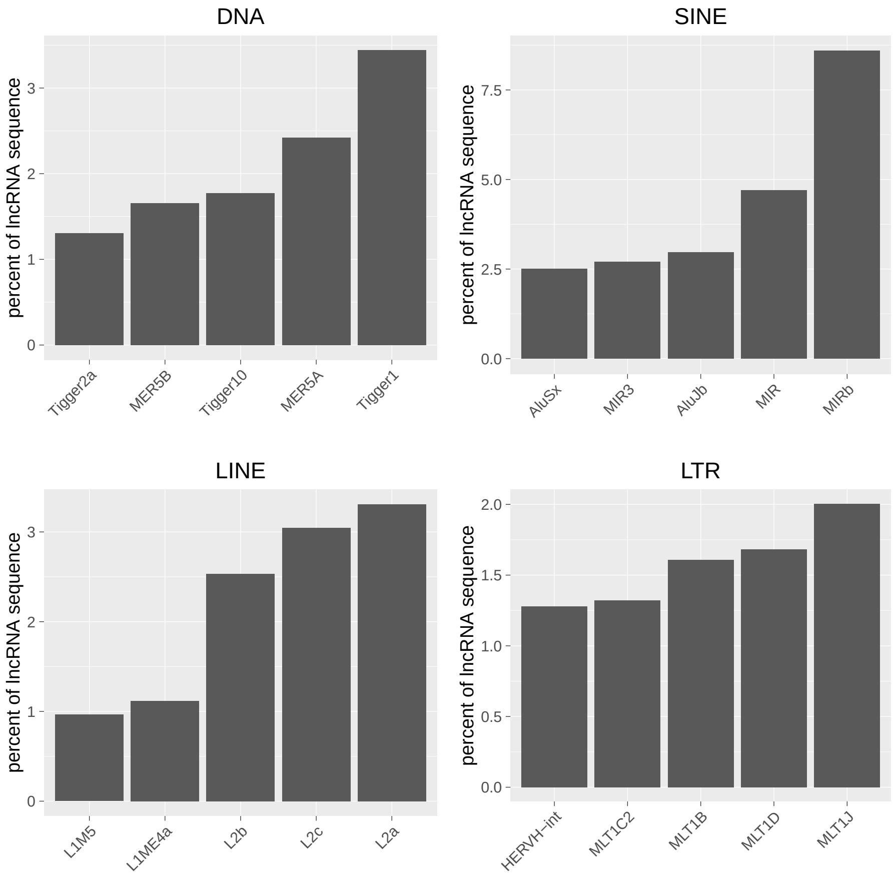

Figure S7: Percent of human lncRNA sequence made up by different TE families. The top 5 families from each of the 4 main classes are shown.

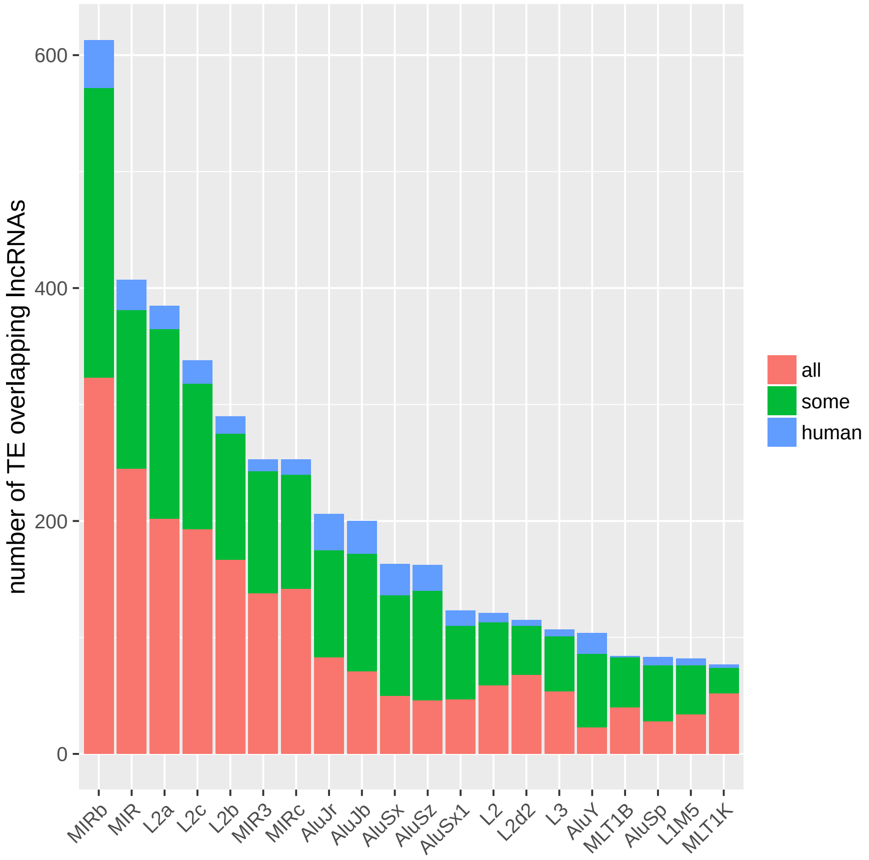

Figure S8: TEs that occur most frequently in human iPSC lncRNAs. Red represents lncRNAs which are conserved in all 4 primate species. Green are those conserved in 1 or 2 other NHPs. Blue are human specific lncRNAs.

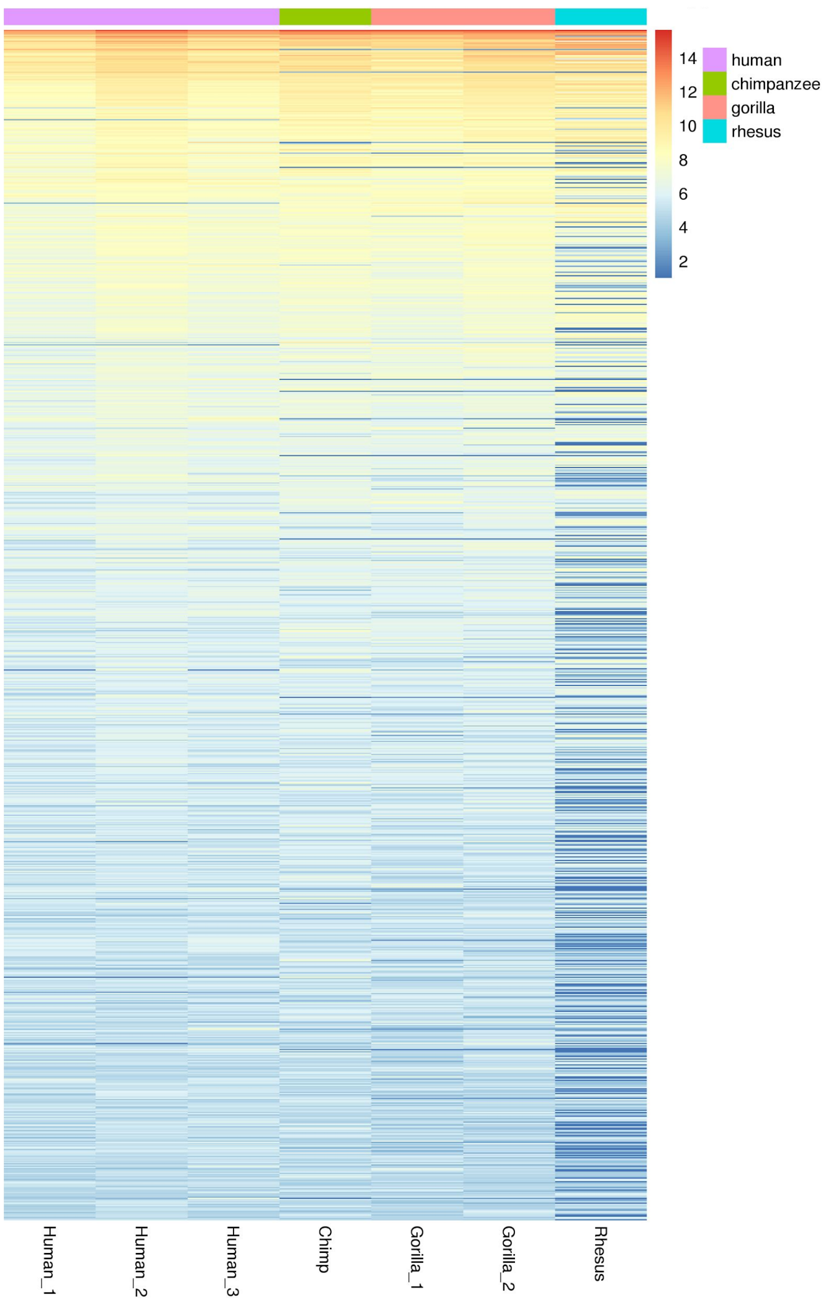

Figure S9: Heatmap of the expression of the top 1000 protein coding genes that are orthologous between human and the 3 NHPs. Coloring represents normalized level of gene expression.

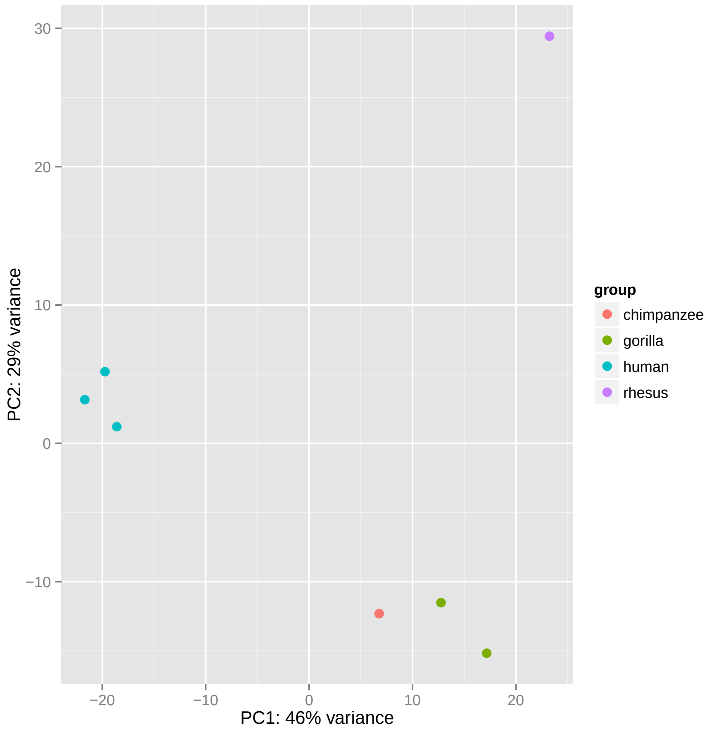

Figure S10: PCA analysis of the expression of all protein coding genes that are orthologous between human and the 3 NHPs.

2 Supplementary Tables

Table S1: Total number of TEs in each species' annotation, and the number of human TEs which have orthologous locations in the NHPs.

**Species**	**TE annotation**	**Conserved**
human (hg19)	4419227	–
chimp (panTro4)	4287630	3984803 (92.9%)
gorilla (gorGor3)	4291825	3913452 (91.2%)
rhesus (rheMac3)	4259993	3597952 (84.5%)

Table S2: HERVH LiftOver verification. The number of LiftOver HERVH that are also annotated as HERVH by RepeatMasker in the target species. There are 1073 HERVH in human that do not overlap coding regions.

**Species**	**LiftOver**	**Properly annotated**
chimp (panTro4)	785	724 (92.2%)
gorilla (gorGor3)	681	615 (90.3%)
rhesus (rheMac3)	157	136 (86.6%)

Table S3: Table of values for Figures 1-2 and table 1. See Supplementary File Table_S3.txt.

Table S4: RNA-seq read statistics

genome (replicate)	raw_reads	filtered_reads	norRNA_reads	aligned_reads
hg19 (1)	95,376,492	90,928,670	83,252,744	79,994,502
hg19 (2)	88,425,668	84,434,790	76,504,782	73,685,598
hg19 (3)	113,399,220	108,111,272	79,458,028	75,815,753
hg19 (merged)	297,201,380	283,474,732	239,215,554	229,495,870
gorGor3 (1)	99,352,516	94,324,612	82,850,012	74,300,557
gorGor3 (2)	152,356,804	144,850,302	107,401,888	96,610,930
panTro4 (1)	111,261,404	105,425,806	90,111,522	84,339,461
rheMac3 (1)	73,458,568	67,867,152	59,762,614	53,060,992

Table S5: Table of coordinates for TEs in table 1. See Supplementary File Table_S5.txt.

Table S6: The number of transcripts in each lncRNA annotation and proportion of transcripts which overlap at least one TE. After *de novo* lncRNA discovery the number of lncRNA transcripts is approximately comparable between human, chimp, and gorilla.

**Species**	**With guide**		***De novo***	
	**# of transcripts**	**Overlap TEs**	**# of transcripts**	**Overlap TEs**
Human	9332	72.80%	1114	80.90%
Chimpanzee	1848	71.80%	1734	74.10%
Gorilla	1323	70.80%	1342	70.60%
Rhesus	882	79.10%	37	75.70%

Table S7: List of biotypes removed from Cufflinks transcript annotation for lncRNA annotation.

**Transcript classification**
**Protein coding**<br>IG_C_gene, IG_D_gene, IG_J_gene, IG_LV_gene, IG_M_gene,<br>IG_V_gene, IG_Z_gene, nonsense_mediated_decay,<br>mathsf nontranslating_CDS, non_stop_decay, polymorphic_pseudogene,<br>protein_coding, TR_C_gene, TR_D_gene, TR_gene, TR_J_gene, TR_V_gene
**Pseudogene**<br>disrupted_domain, IG_C_pseudogene, IG_J_pseudogene,<br>IG_pseudogene, IG_V_pseudogene, processed_pseudogene, pseudogene,<br>transcribed_processed_pseudogene, transcribed_unprocessed_pseudogene,<br>translated_processed_pseudogene, translated_unprocessed_pseudogene,<br>TR_J_pseudogene, TR_V_pseudogene, unitary_pseudogene, unprocessed_pseudogene
**Short non-coding**<br>miRNA, miRNA_pseudogene, misc_RNA, misc_RNA_pseudogene,<br>Mt_rRNA, Mt_tRNA, Mt_tRNA_pseudogene, ncRNA, pre_miRNA, RNase_MRP_RNA,<br>RNase_P_RNA, rRNA, rRNA_pseudogene, scRNA_pseudogene, snlRNA, snoRNA,<br>snoRNA_pseudogene, snRNA, snRNA_pseudogene, SRP_RNA, tmRNA, tRNA, tRNA_pseudogene
